# Biological aging mediates the associations between urinary metals and osteoarthritis among U.S. adults

**DOI:** 10.1186/s12916-022-02403-3

**Published:** 2022-06-17

**Authors:** Li Chen, Ying Zhao, Fangqu Liu, Huimin Chen, Tianqi Tan, Ping Yao, Yuhan Tang

**Affiliations:** Department of Nutrition and Food Hygiene, Hubei Key Laboratory of Food Nutrition and Safety, Ministry of Education Key Laboratory of Environment and Health and MOE Key Lab of Environment and Health, Key Laboratory of Environment and Health (Wuhan), Ministry of Environmental Protection, State Key Laboratory of Environment Health (Incubation), School of Public Health, Tongji Medical College, Huazhong University of Science and Technology, Wuhan, 430030 China

**Keywords:** Heavy metals, Osteoarthritis, Joint exposure, Biological aging, Mediation

## Abstract

**Background:**

Osteoarthritis (OA) is a worldwide public health concern, mainly afflicting older adults. Although the etiology of OA remains unclear, environmental factors are increasingly considered as non-negligible risk factors. This study aims to evaluate the associations of urinary metals with OA risk and the mediated effect of biological aging.

**Methods:**

Nine urinary metal concentrations were detected among 12,584 U.S. adults based on the National Health and Nutrition Examination Survey (NHANES), including barium (Ba), cadmium (Cd), cobalt (Co), cesium (Cs), molybdenum (Mo), lead (Pb), antimony (Sb), thallium (Tl), and uranium (Tu). Multivariable logistic regression and weighted quantile sum (WQS) regression were used to explore the associations of single metal and mixed metals with OA risk, respectively. Furthermore, biological aging was measured from different perspectives, including cell senescence (telomere length) and whole-body aging (phenotypic age and biological age). Mediation analyses were conducted to investigate the mediated effects of aging on the associations of metals with OA risk.

**Results:**

In the single-exposure model, Cd, Co, and Cs were identified to be positively associated with OA risk, with odds ratios (OR) ranging from 1.48 to 1.64 (all *P* < 0.05). Mixed-exposure analyses showed consistent associations (OR 1.23, 95%CI 1.10 to 1.37) and highlighted that Cd, Co, and Cs were responsible for the outcomes. Additionally, Cd, Co, Cs, Pb, and Tl were positively associated with biological aging markers, while all biological aging markers had significant associations with OA risk. Further mediation analyses showed that the associations of single metal (mainly Cd and Cs) and mixed metals with OA risk parallelly mediated by the above biological aging markers, with the proportion of mediation ranging from 16.89 to 69.39% (all *P* < 0.05). Moreover, such associations were also serially mediated through telomere length-biological age path and telomere length-phenotypic age path (the proportion of mediation: 4.17–11.67%), indicating that metals accelerated cell senescence to lead to whole-body aging and finally aggravated OA progress.

**Conclusions:**

These findings suggested that exposure to metals increased OA risk, which was possibly and partly mediated by biological aging.

**Supplementary Information:**

The online version contains supplementary material available at 10.1186/s12916-022-02403-3.

## Background

Osteoarthritis (OA) is a joint disorder with progressive cartilage breakdown, subchondral remodeling, and synovial inflammation. With an increasingly aging population, this syndrome is becoming a worldwide public health concern and a leading source of societal cost in older adults [1]. Epidemiologically, the global age-standardized point prevalence of OA was 3.75% in 2017 and the age-standardized prevalence from 1990 to 2017 was increased by 23.2% in the USA [2]. It is estimated that the number of U.S. people afflicted by OA will rise to 67 million by 2030 [3, 4]. Although the etiology of OA has not been fully elucidated, the non-negligible environmental factors are growingly considered to account for this disease.

Heavy metals are ubiquitously present in various environmental media, such as air, soil, drinking water, and food [5, 6]. The widespread exposure to toxic metals leads to illnesses, such as diabetes [7] and cancer [8]. Of concern, heavy metals (e.g., cadmium (Cd) and lead (Pb)) were cumulative bone-toxicants with the contribution to musculoskeletal disease [9]. Compelling toxicological evidence showed that heavy metals were linked to two major pathological changes of OA, including joint damage and inflammation [10, 11]. For example, cadmium chloride treatment caused cartilage loss and inflammation in a 3D culture model of human chondrocytes [12]. Also, a cross-sectional study showed blood Pb was significantly associated with knee OA in Korean postmenopausal women [13]. Previous epidemiological researches have presented positive associations of heavy metals with rheumatoid arthritis [14] and osteoporosis [15]. However, population study on the impact of heavy metals on OA is limited. Moreover, the role of metals in diseases usually relied on their cooperation and interactions. Individual metals cannot completely account for the occurrence and development of disease. Hence, it is worthwhile to explore the joint effects of metals on OA. Additionally, underlying mechanisms of metals in OA remain largely unrevealed.

Aging, a progressive decline in body homeostasis, is considered a target for metal action. Toxicologically, heavy metals can render cell senescence and body premature aging by mitochondrial dysfunction, oxidative stress, and DNA damage [16, 17]. Epidemiological research also reported the positive associations of Cd and Pb with telomere wear [18]. On the other hand, aging is the major pathogenesis for OA. Researchers emphasized that joint tissues are subject to aging and decay over time and the senescence of joint homeostasis-related cells contributed to the development of OA [19]. Cohort studies also showed a negative correlation between telomere length and OA incidence [20]. Importantly, elimination of senescent chondrocytes improved joint degeneration in OA mice, indicating biological aging was responsible for OA development [21]. Collectively, based on the importance of biological aging for metal exposure and OA respectively, we hypothesized that heavy metal exposure may increase OA risk by promoting biological aging.

Hence, we conducted a cross-sectional study to investigate the associations of 9 urinary metals with OA risk based on the National Health and Nutrition Examination Survey (NHANES) 1999–2016. Further, we measured biological aging status at multiple perspectives and explored the mediated effects of biological aging.

## Methods

### Study population

**Fig. 1:**
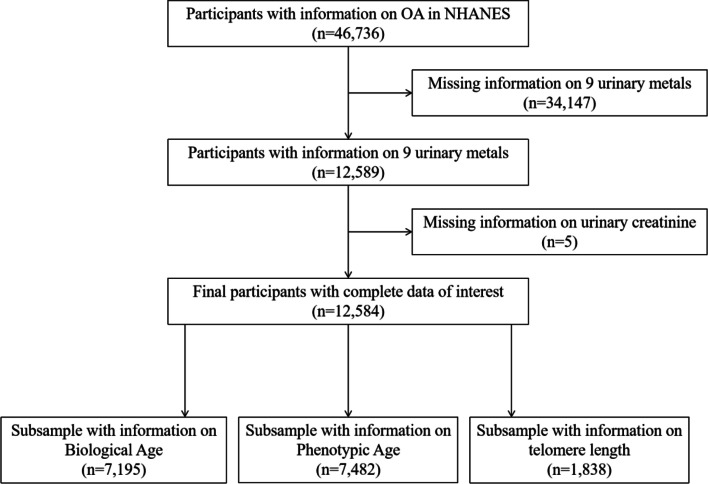
Flow chart of participants selection. NHANES, National Health and Nutrition Examination Survey

The NHANES is an ongoing national cross-sectional survey and data is available on the website of the America Centers for Disease Control and Prevention (CDC) (http://www.cdc.gov/nchs/nhanes.htm). The study protocol was approved by the National Center for Health Statistics (NCHS) Research Ethics Review Board. All participants provided written consent at the time of recruitment. In the current study, OA patients were included according to self-reported information (*n* = 46,736). Next, we excluded individuals whose information on 9 metals was missing (*n* = 34,147). Further, we excluded individuals with missing creatinine data (*n* = 5). Collectively, 12,584 participants were enrolled, including 1356 OA patients (Fig. 1).

### OA assessment

A study showed an 81% agreement between self-report and clinical confirmation of OA [22]. In NHANES, OA was diagnosed by professionals and the information was collected by questionnaire survey. Briefly, all participants ≥ 20 years of age were asked two arthritis-related questions. First, they were inquired: “Has a doctor or other health professional ever told you that you have arthritis?” Individuals with an affirmative response were asked a follow-up question: “Which type of arthritis was it?” The participants who answered “osteoarthritis” were enrolled in the study.

### Metal measurement

The detection data of 9 urinary metals were obtained from NHANES 1999–2016. Barium (Ba), Cd, cobalt (Co), cesium (Cs), molybdenum (Mo), Pb, antimony (Sb), thallium (Tl), and uranium (Tu) were primarily measured from spot urine samples by using inductively coupled plasma mass spectrometry (ICP-MS). The limit of detection (LOD) divided by the square root of two was used to replace the values below the LOD. The concentrations of metals were all corrected by urinary creatinine and were expressed as micrograms per gram creatinine.

### Measurement of biological aging markers

Biological age and phenotypic age used different calculation methods and incorporated different biomarkers to measure biological aging. Biological age was proposed by Klemera and Doubal [23] based on 8 biomarkers (Ln-C-reactive protein (CRP), serum creatinine, glycosylated hemoglobin, serum albumin, serum total cholesterol, serum urea nitrogen, serum alkaline phosphatase, and systolic blood pressure) [24]. The values *j* and *i* represent the number of biomarkers and samples respectively. The values *k*, *q*, and *s* are the regression slope, intercept, and the root means squared error of a biomarker regressed on chronological age, respectively. The value *r*_j_^2^ represents the variance explained by regression of chronological age on biomarkers.

1 $${\text{BA}}_{E}=\frac{{\sum }_{j=1}^{m}\left({x}_{j}-{q}_{j}\right)\left(\frac{{k}_{j}}{{s}_{j}^{2}}\right)}{{\sum }_{j=1}^{m}{\left(\frac{{k}_{j}}{{s}_{j}}\right)}^{2}}$$

2 $${r}_{char}=\frac{{\sum }_{j=1}^{m}\frac{{r}_{j}^{2}}{\sqrt{1-{r}_{j}^{2}}}}{{\sum }_{j=1}^{m}\frac{{r}_{j}}{\sqrt{1-{r}_{j}^{2}}}}$$

3 $${s}_{BA}^{2}={\frac{{\sum }_{j=1}^{n}\left(\left({\text{BA}}_{Ei}-{\text{C}}{\text{A}}_{i}\right)- \frac{{\sum }_{i=1}^{n}\left({\text{BA}}_{Ei}-{\text{C}}{\text{A}}_{i}\right)}{n}\right)}{n}}^{2}-\left(\frac{1-{r}_{char}^{2}}{{r}_{char}^{2}}\right)\times \left(\frac{{\left({\text{CA}}_{max}- {CA}_{min}\right)}^{2}}{12m}\right)$$

4 $$\mathrm{Biological\ age}=\frac{{\sum }_{j=1}^{m}\left({x}_{j}-{q}_{j}\right)\left(\frac{{k}_{j}}{{s}_{j}^{2}}\right)+ \frac{\text{CA}}{{s}_{BA}^{2}}}{{\sum }_{j=1}^{m}{\left(\frac{{k}_{j}}{{s}_{j}}\right)}^{2}+ \frac{1}{{s}_{\text{BA}}^{2}}}$$

Phenotypic age was calculated by using 9 aging-related variables [25], including chronological age, albumin, creatinine, glucose, CRP, lymphocyte percent, mean cell volume, red blood cell distribution width, alkaline phosphatase, and white blood cell count.

$$\mathrm{Phenotypic\ age} =141.50 +\frac{\mathrm{Ln}[-0.00553 \times \mathrm{Ln}(\mathrm{exp}(\frac{-1.51714\times \mathrm{exp}(\mathrm{xb})}{0.0076927}))]}{0.09165}$$

where xb =  − 19.907 − 0.0336 × Albumin + 0.0095 × Creatinine + 0.1953 × Glucose + 0.0954 × LnCRP-0.0120 × Lymphocyte Percent + 0.0268 × Mean Cell Volume + 0.3306 × Red Cell Distribution Width + 0.00188 × Alkaline Phosphatase + 0.0554 × White Blood Cell Count + 0.0804 × Chronological Age.

The measurement method of telomere length has been reported elsewhere [26]. Briefly, blood samples were collected and determined by quantitative polymerase chain reaction (qPCR) assays to assess the telomere length relative to standard reference DNA (the T/S ratio). All sample analyses were performed in duplicate strictly following the manufacturer’s instruction and all results were checked to meet the laboratory’s standardized criteria for acceptability before being released for reporting.

### Statistical analysis

All analyses were performed with SAS (version 9.4) or R (version 3.6.3) and accounted for the complex sampling design of the NHANES, and the code for analyses were in Additional file 2. Chi-square tests and *t*-tests were used to assess participants’ demographic characteristics by OA status. The metal concentrations were Ln-transformed to obtain approximate normal distribution (continuous variables) or categorized into four quartiles (Q1, Q2, Q3, and Q4) as categorical variables. Multivariable logistic regression was applied to estimate odds ratios (OR) and corresponding 95% confidence interval (CI) for the associations of metals and biological aging markers with OA risk. Multivariable linear regression was used to explore the associations of metals with biological aging markers. All analyses adjusted covariates for age (< 60 or ≥ 60 years), sex (male or female), race/ethnicity (Mexican American, other Hispanic, non-Hispanic White, non-Hispanic Black, or others), marital status (married/cohabiting, widowed/divorced/separated, or never married), physical activity (moderate or vigorous), drinking alcohol status (ever or never), body mass index (BMI), the ratio of family income to poverty (PIR), and serum cotinine concentration. Missing data for the covariates were coded as a missing indicator category for categorical variables and were imputed with the median for the continuous variable. We used the false-discovery rate (FDR) correction to adjust for multiple tests in the regression models. The trend test across increasing exposure groups was calculated using integer values (1, 2, 3, and 4). Pearson correlation analysis was used to assess the correlations among Ln-transformed metals. The directed acyclic graphs (R package “dagitty” and “ggdag”) showed the main relationships among exposures, outcomes, covariates, and mediators (Additional file 1: Fig. S1).

Weighted quantile sum (WQS) regression was applied to explore the overall effects of metals on OA as it performed well in characterizing environmental mixtures [27]. R package (“gWQS”) can empirically calculate the WQS index comprised of weighted sums of individual metal concentrations. The WQS index (ranged from 0 to 1) represented the mixed exposure level of metals, and the components of concern were identified by non-negligible weights. The final result was interpreted as the simultaneous effect on OA of a one-quantile increase of mixed metals.

Several sensitivity analyses were also performed. First, given the potential non-linearity and non-additive relations among metals, Bayesian kernel machine regression (BKMR) was used to assess the joint effect of all metals (“BKMR” packages) and the dose–response relationship between single metal and OA risk when fixing other metal concentrations. Second, we further adjusted for the occupation of participants (industry and agriculture, service and transportation, and others). Third, we further adjusted for age-related diseases including diabetes (yes or no), hypertension (yes or no), cardiovascular disease (yes or no) and cancer (yes or no), and OA-related medicine use (e.g., meloxicam, celecoxib) (yes or no). Fourth, we further adjusted for the survey cycle (1, 2, 3, 4, 5, 6, 7, 8, and 9). Fifth, participants with abnormal urinary creatinine were excluded. Finally, pregnant participants were excluded.

The potential mediating effects of biological aging markers on the associations of single and mixed metals (shown as WQS index) with OA risk were estimated by two mediation models, including parallel mediation (R package “mediation”) and serial mediation analyses (R package “bruceR”). Parallel mediation models used individual indicators as a mediator, while serial mediation models used a pathway as a mediator. Mediation analyses used the quasi-Bayesian Monte Carlo method with 1000 simulations based on normal approximation. The direct effect (DE) represented the effects of metal exposure on OA without a mediator. The indirect effect (IE) represented the effects of metal exposure on OA through the mediator. The proportion of mediation was calculated by using IE divided by TE (total effect).

## Results

### Characteristics of participants and metals distribution

Among the 12,584 adults, 1356 were diagnosed with OA. The demographic characteristics of the study participants with or without OA are listed in Table 1. Overall, age, sex, ethnicity, marital status, physical activity, drinking alcohol status, family PIR, BMI, serum cotinine, biological age, phenotypic age, and telomere length were statistically significant between OA and non-OA participants.

**Table 1:** Basic characteristics of participants by OA among U.S. adults, NHANES 1999–2016

Characteristics	Total (12,584)	OA (1356)	Non-OA (11,228)	*P* value
Age, *n* (%)				< 0.001
20–59	8981 (79.39)	457 (42.56)	8524 (84.01)	
≥ 60	3603 (20.60)	899 (57.44)	2704 (15.99)	
Sex, *n* (%)				< 0.001
Male	6234 (48.89)	498 (36.51)	5736 (50.44)	
Female	6350 (51.11)	858 (63.49)	5492 (49.56)	
Race/ethnicity, *n* (%)				< 0.001
Mexican American	2270 (8.53)	105 (2.49)	2165 (9.30)	
Other Hispanic	1059 (5.63)	99 (2.80)	960 (5.98)	
Non-Hispanic White	5595 (68.30)	887 (84.21)	4708 (66.30)	
Non-Hispanic Black	2540 (10.91)	193 (6.10)	2347 (11.51)	
Other race/multiracial	1120 (6.63)	72 (4.40)	1048 (6.91)	
Education, *n* (%)				0.857
Under high school	3263 (16.84)	327 (16.71)	2936 (16.86)	
High school or equivalent	2895 (23.66)	317 (22.95)	2578 (23.75)	
Above high school	6416 (59.50)	711 (60.35)	5705 (59.39)	
Marital status, *n* (%)				< 0.001
Married/cohabiting	7725 (64.34)	780 (65.16)	6945 (64.23)	
Widowed/divorced/separated	2401 (16.62)	464 (27.88)	1937 (15.20)	
Never married	2368 (19.04)	105 (6.96)	2263 (20.56)	
Physical activity, *n* (%)				< 0.001
Moderate	8239 (59.89)	1076 (75.85)	7163 (57.89)	
Vigorous	4343 (40.11)	279 (24.15)	4064 (42.11)	
Drinking alcohol status, *n* (%)				0.001
Never	8174 (76.31)	870 (71.12)	7304 (76.98)	
Ever	3333 (23.69)	416 (28.85)	2917 (23.02)	
Family PIR	3.01 ± 0.03	3.12 ± 0.07	3.00 ± 2.93	0.013
BMI (kg/m^2^)	28.37 ± 0.10	30.66 ± 0.28	28.09 ± 0.10	< 0.001
Serum cotinine (ng/mL)	57.15 ± 1.86	50.0 ± 4.62	58.06 ± 1.99	0.036
Biological age (years)	44.92 ± 0.31	60.91 ± 0.65	43.24 ± 0.30	< 0.001
Phenotypic age (years)	40.17 ± 0.33	57.45 ± 0.67	38.34 ± 0.31	< 0.001
Telomere length	1.07 ± 0.01	0.95 ± 0.03	1.09 ± 0.01	< 0.001

Continuous variables were presented as mean ± SE. Categorical variables were presented as *n* (%). *NHANES* National Health and Nutrition Examination Survey, *OA* osteoarthritis, *PIR* the ratio of family income to poverty, *BMI* body mass index, *SE* standard error, *n* numbers of subjects; %, weighted percentage

The distribution of metal concentrations is shown in Additional file 1: Table S1. The detection rates of metals were greater than 75%. Pearson’s coefficient between Ln-transformed metals showed moderate correlations between Cs and Tl (*r* = 0.58), Ba and Co (*r* = 0.41), and Cd and Pb (*r* = 0.40), while other correlations were relatively poor (Additional file 1: Fig. S2).

### Associations between metal concentration and OA risk

**Fig. 2:**
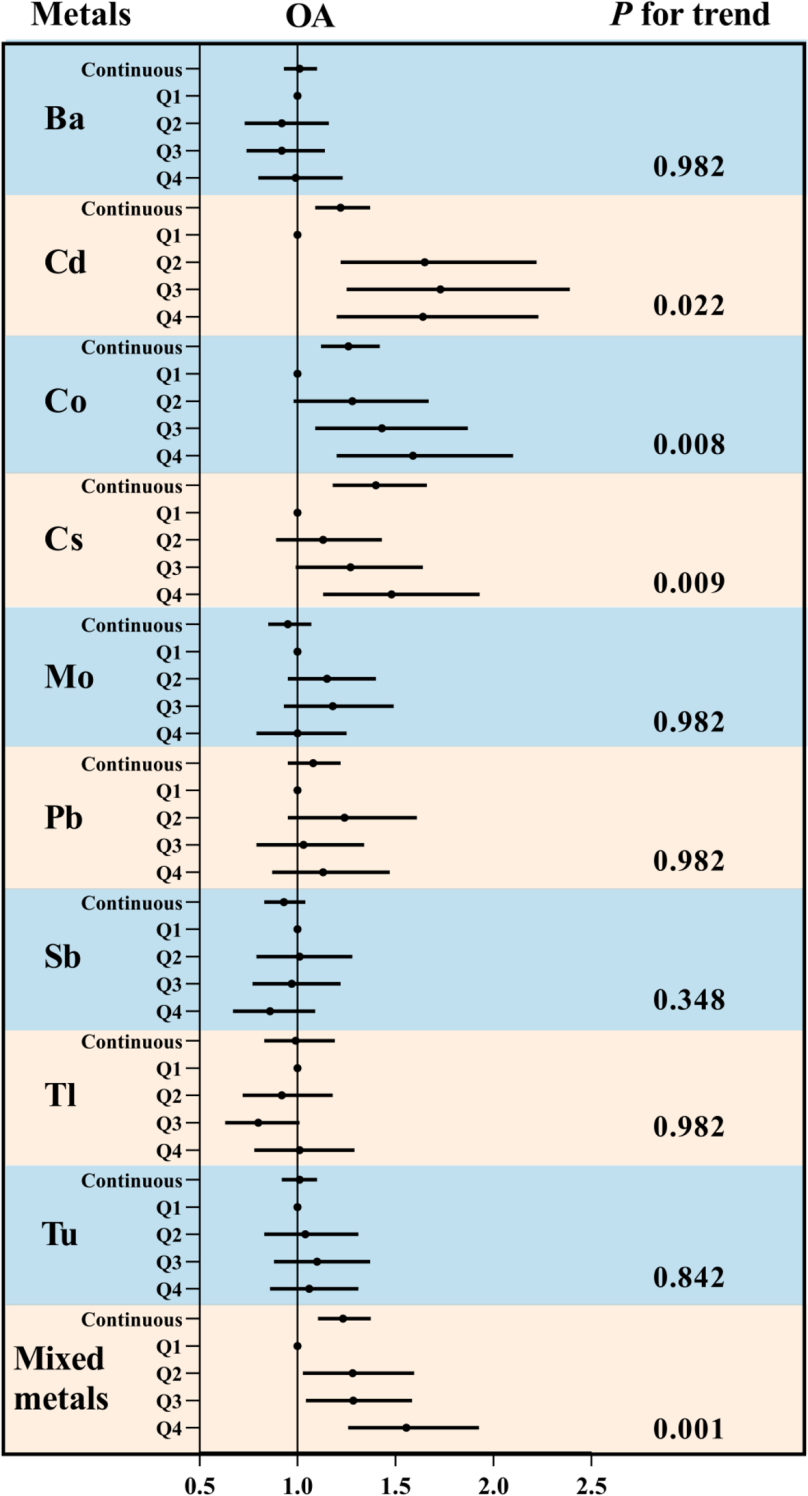
OR (95% CI) in OA associated with single and mixed metals levels. Models were adjusted for sex, age, race/ethnicity, education, family income-to-poverty ratio, marital status, body mass index, physical activity, drinking alcohol status, and serum cotinine. Continuous, Ln-transformed concentration of metals; Q, quartile; OA, osteoarthritis. All *P* for trend were FDR-adjusted

Figure 2 displayed the associations between creatinine-adjusted metal concentrations and OA risk by the survey-weighted logistic regression models. The highest exposure quantile of Cd (OR 1.64, 95%CI 1.20 to 2.23), Co (OR 1.59, 95%CI 1.20 to 2.10), and Cs (OR 1.48, 95%CI 1.13 to 1.93) increased the risk of OA compared to quantile 1 (all *P* for trend < 0.05). These associations were also presented in Ln-transformed metals and OA risk (all *P* < 0.05).

The WQS index of mixed metals was positively associated with OA risk (OR 1.23, 95%CI 1.10 to 1.37) (Fig. 2). Additionally, the highest weighted metals in WQS models were Cd (54.45%), Co (27.14%), and Cs (9.23%) (Additional file 1: Fig. S3).

In the sensitivity analyses, the BKMR model also showed a significant and positive association of mixed metals with OA risk (Additional file 1: Fig. S4). After further adjusting for the occupation of the participants, adjusting for diabetes, hypertension, cardiovascular disease, cancer, and OA-related medicine use, adjusting for the survey cycle, excluding participants with abnormal urinary creatinine, or excluding pregnant participants, the results were not materially changed (Additional file 1: Table S2-6).

### Associations between metal concentration and biological aging markers

**Fig. 3:**
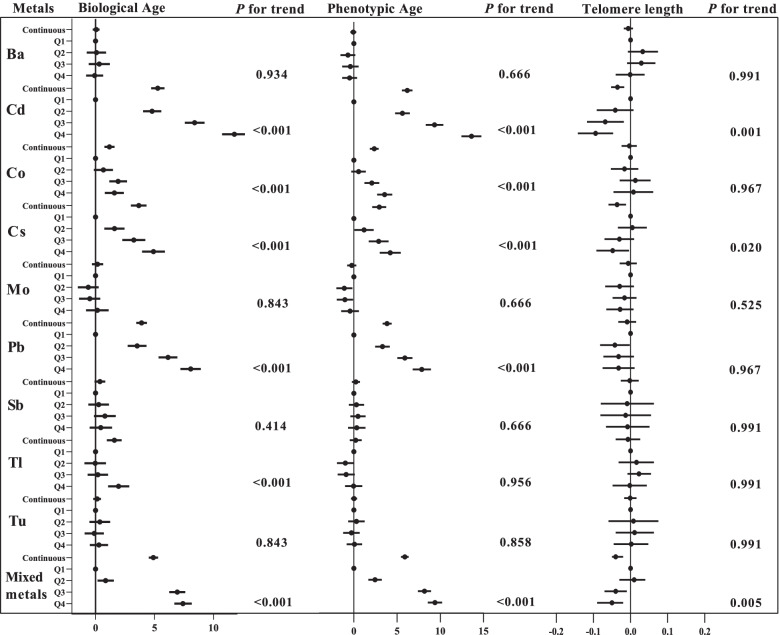
Regression coefficients (95% CI) in aging markers associated with single and mixed metal levels. Models were adjusted for sex, age, race/ethnicity, education, family income-to-poverty ratio, marital status, body mass index, physical activity, drinking alcohol status, and serum cotinine. Continuous, Ln-transformed concentration of metals; Q, quartile. All *P* for trend were FDR-adjusted

Figure 3 showed the associations of metals with biological aging markers based on the linear regression. We found the highest quartile of Cd, Co, Cs, Pb, and Tl (versus quartile 1) were associated with a growing of biological age (all *P* for trend < 0.001). With increasing quantiles of Cd, Co, Cs, and Pb, phenotypic age was increased (all *P* for trend < 0.001). Cd and Cs were negatively associated with telomere length (all *P* for trend < 0.05). Moreover, mixed metals had positive associations with biological age (*β* 4.91, 95%CI 4.52 to 5.31) and phenotypic age (*β* 5.90, 95%CI 5.45 to 6.34) and had a negative association with telomere length (*β* − 0.04, 95%CI − 0.05 to − 0.02).

### Associations between biological aging markers and OA risk

Table 2 shows the associations of aging markers with OA risk based on the logistic regression. Each 1-year increase in biological age increased the risk of OA by 6% (95%CI 1.05 to 1.07). Similarly, each 1-year increase in phenotypic age was associated with an increased OA risk (OR 1.04, 95%CI 1.04 to 1.05). Additionally, for each unit increase in telomere length (mean T/S ratio), OR for OA decreased by 74% (95%CI 0.07 to 0.97), which was consistent with the quantile analysis (Q4 vs. Q1: OR 0.40, 95%CI 0.17 to 0.90).

**Table 2:** Associations of aging makers with OA risk, NHANES 1999–2016

	Continuous	Q1	Q2	Q3	Q4	*P* for trend
	OR (95% CI)		OR (95% CI)	OR (95% CI)	OR (95% CI)	
Biological age	1.06 (1.05, 1.07)	1.00 (reference)	4.32 (1.90, 9.84)	15.1 (6.69, 34.07)	16.75 (7.46, 37.64)	< 0.001
Phenotypic age	1.04 (1.04, 1.05)	1.00 (reference)	6.48 (2.74, 15.31)	18.78 (8.03, 43.93)	22.91 (9.31, 56.37)	< 0.001
Telomere length	0.26 (0.07, 0.97)	1.00 (reference)	0.76 (0.37, 1.54)	0.58 (0.30, 1.11)	0.40 (0.17, 0.90)	0.022

Models were adjusted for sex, age, race/ethnicity, education, family income-to-poverty ratio, marital status, body mass index, physical activity, drinking alcohol status, and serum cotinine. Q, quartile. All *P* for trend were FDR-adjusted

### Mediation analyses

**Fig. 4:**
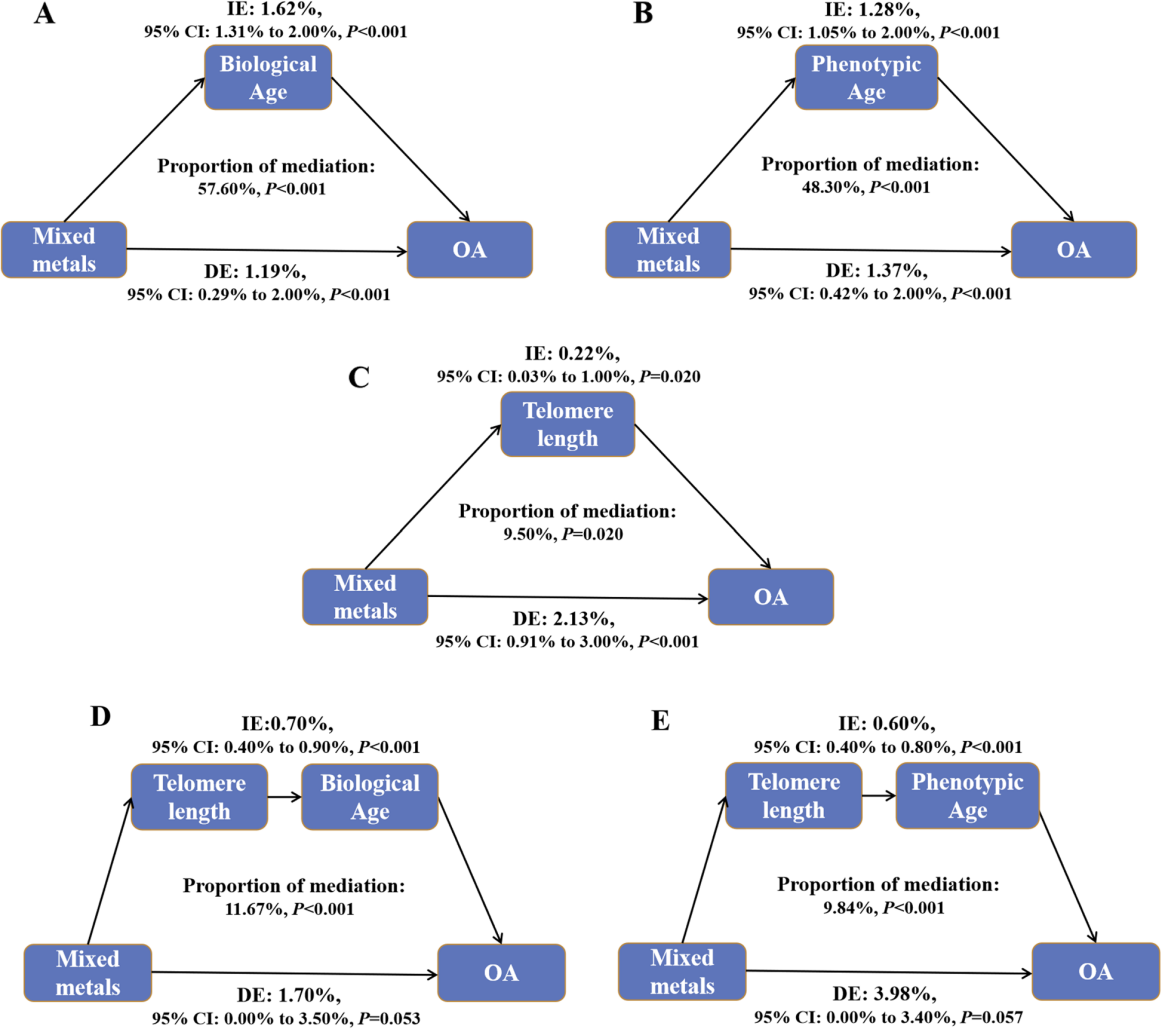
Estimated proportion of the association between urinary mixed metals and OA mediated by biological age (**A**), phenotypic age (**B**), and telomere length (**C**) and serially mediated by telomere length-biological age path (**D**) and telomere length-phenotypic age path (**E**). Models were adjusted for sex, age, race/ethnicity, education, family income-to-poverty ratio, marital status, body mass index, physical activity, drinking alcohol status, and serum cotinine. IE, the estimate of the indirect effect; DE, the estimate of the direct effect; Proportion of mediation = IE/DE + IE

Furthermore, parallel mediation analyses were performed to evaluate the potential mediation effects of biological aging on the associations of metals with OA risk. Biological age had significant mediated effects on the associations of Cd, Co, and Cs with OA risk, and the proportion of mediation was 69.27%, 18.43%, and 30.50% respectively (all *P* < 0.05). The mediated proportion of phenotypic age was 61.01%, 31.83%, and 17.00% on the associations of Cd, Co, and Cs with OA risk respectively. Telomere length also mediated the association between Cs and OA risk and the proportion of mediation was 9.81% (Additional file 1: Table S7). Moreover, biological age, phenotypic age, and telomere length parallelly mediated the associations of mixed metals with OA risk with 57.60%, 48.30%, and 9.50% proportion of mediation respectively (all *P* < 0.05) (Fig. 4).

As cell senescence is a leading cause of whole-body aging, we further explored the potential paths of associations between metals and OA risk by the serial mediation models. Additional file 1: Table S8 shows the association between Cs and OA risk serially mediated by telomere length-biological age path and telomere length-phenotypic age path, with 4.55% and 4.17% proportion of mediation respectively. Besides, the serial mediated effect of telomere length-biological age path and telomere length-phenotypic age path on the association of mixed metals with OA risk were 0.70% and 0.60% respectively. And the proportion of mediation was 11.67% and 9.84%, respectively (Fig. 4).

## Discussion

In the current study, we mainly provided two novel findings based on the U.S. general population. First, the single and mixed exposure models consistently emphasized Cd, Co, and Cs as strong risk factors for OA. Furthermore, biological aging was identified as a mediator for the positive associations of metals, especially mixed metals, with OA risk.

Most toxic metals are widely distributed in industrial products, soil, food, and drinking water and mainly accumulated in the liver, kidney, muscle, and bone [28, 29]. Compared to patients who suffered other bone injuries, OA patients had a higher heavy metal concentration in bone, such as Cd and Co [30]. Moreover, the toxicological study showed that Cd exposure rendered bone or joint-related injuries, such as cartilage loss [9]. Our study firstly provided epidemiological evidence on the positive and significant associations of Cd, Co, and Cs with OA risk. Similarly, our recent cross-sectional study suggested a significant association between Cd and rheumatoid arthritis risk, further indicating Cd was responsible for musculoskeletal disorders [14]. Studies of other models also provided supportive evidence of metals causing inflammation. For example, Cd, Co, or Cs exposure triggered inflammation with the upregulation of IL-6 in mice brain, heart, or macrophages [31–33].

Noteworthy, heavy metals usually coexist in the environment and their effects rely on their cooperation and interaction. Previous studies reported that the mixture of Cd and Pb further diminished skeletal growth and caused osteopenia compared to single metals in rats. The mixed exposure is measured by WQS with an empirically weighted index in the direction of the joint action, in which components of concern are identified by non-negligible weights. This method truly reflects the joint action of mixed exposure. In our study, Cd, Co, and Cs were identified as the strongest risk factors for OA. Also, mixed metals were positively associated with OA risk in WQS models and BKMR models, further suggesting that metal exposure may promote OA progress.

Mechanistically, our research interest focused on biological aging as a growing number of studies emphasized the role of metals in age-related diseases [34]. Our results showed that both single (Cd, Co, Cs, Pb, and Tl) and mixed metals were positively associated with biological aging. Consistent toxicological evidence presented that heavy metals can induce senescence in stromal and vascular smooth muscle cells (e.g., Co and Cd) [35, 36], as well as accelerate brain aging in animals, shown by damaged molecules and impaired energy metabolism [17]. Also, a recent population study indicated the positive associations between heavy metals (e.g., Co) and biological aging marker (DNA methylation age) [37]. Many investigations on metals and telomere length have reached similar results [38]. In addition, heavy metals may regulate senescence-related pathways, including Keap1-NRF2, nuclear translocation of NF-κB, and the high expression of HIF [11]. Collectively, the abovementioned evidence supported our finding that heavy metals may modulate aging.

On the other hand, aging is one of the crucial drivers for chronic diseases, especially OA. Aging promoted OA progress mainly through the accumulation of senescent cells, senescence-related secretory phenotype (SASP) production, and ROS-induced DNA damage [19]. Herein, we measured cellular senescence and whole-body aging using different markers and consistently found positive associations between biological aging and OA risk. Specifically, phenotypic age and biological age incorporate composite clinical biomarkers, such as inflammation (e.g., CRP), immunity (e.g., number of immune cells), and organ function (e.g., albumin and serum urea nitrogen). Both of which are useful to identify the unhealthy condition of people caused by multiple system disorders [39]. Interestingly, OA is a whole-joint disease accompanied by system inflammation, indicating the significance of whole-body aging in OA. Additionally, telomere wear is widely used to evaluate cell senescence [40]. Abundant epidemiological studies have reported the inverse associations of telomere length with OA risk, which was consistent with our findings [41]. Similarly, experimental studies reported that targeting the elimination of senescent chondrocytes or inhibiting SASP improved OA-related outcomes [21, 42]. Overall, aging is an important biological mechanism of OA onset and progress.

According to the mentioned findings, we further performed mediation analyses. We observed significant mediated effects of aging markers on the associations of metals with OA risk. Notably, three biological aging markers all mediated the positive association between Cs and OA risk, among which the mediated proportion of biological age was 30.50%, phenotypic age was 17.00%, and telomere length was 9.81%. In fact, Cs radiation has long been considered an inducer of premature aging in animals and can cause bone loss [43, 44]. What is more, the associations of mixed metals with OA risk are also mediated by these aging markers. More importantly, the mediated proportion of whole-body aging was more than 50%, indicating it was largely involved in the promoting process of metals on OA.

Interestingly, whole-body aging is mainly driven by cell senescence [45]. For example, transplantation of senescent cells into young mice caused a decline in whole-body function [46]. Inversely, anti-senescence therapeutics (e.g., senolytics and senomorphics) offered good effects on age-related diseases, such as idiopathic pulmonary fibrosis and diabetic kidney disease [47, 48]. Hence, we further explored the underlying path from metals to OA. Serial mediation analyses showed the associations of Cs and mixed metals with OA were mediated by telomere length-biological age path, and telomere length-phenotypic age path, with the mediated proportion ranging from 4.17 to 11.67%. These findings suggested that metals may accelerate cell senescence to lead to overall disorder of the body and finally aggravate OA progress.

This study has some strengths. First, we applied a variety of methods to explore the relationships between metals and OA risk in a relatively large population. What is more, we measured biological aging status from multiple perspectives. There are also limitations. First, a self-reported OA diagnosis may decrease the credibility of the results. Second, single-point measurements of metals did not fully reflect individual real exposure levels. Third, we only analyzed the correlation between 9 metals and OA risk, and further analyses were needed to identify other metals associated with OA risk. Fourth, residual and unmeasured confounders and measurement errors may bias our analyses. Fifth, although we adjusted for the survey cycle, the time span of our analysis was too wide to potentially bias the results. Finally, we conducted mediation analysis in a cross-sectional study design and it was difficult to infer a causal association. Given the limitations in the current study, these results need to be interpreted with caution and further investigations are needed to support our findings.

## Conclusions

In summary, our results presented the positive associations of single and mixed metals with increased OA risk, primarily driven by Cd, Co, and Cs. Additionally, we found that metal exposure was related to aging, and aging was related to OA risk. Furthermore, mediation analyses showed that the associations of metals with OA risk may be mediated by biological aging and serially mediated by cell senescence-whole-body aging path. These findings identified risk factors for OA and indicated aging as an underlying mechanism of metal’s adverse effect on OA.

## Supplementary Information

**Additional file 1: Fig. S1.** Directed acyclic graph. **Fig. S2.** Pearson’s correlation matrix. **Fig. S3.** Weighted values of urinary metals for OA in WQS models. **Fig. S4.** Associations of the urinary metals with OA risk estimated by Bayesian Kernel Machine Regression (BKMR). **Table S1.** Distributions of metals in the study population. **Table S2.** OR (95% CI) in OA associated with single and mixed urinary metals levels with further adjustment for occupation. **Table S3.** OR (95% CI) in OA associated with single and mixed urinary metals levels with further adjustment for other diseases and medicine use. **Table S4.** OR (95% CI) in OA associated with single and mixed urinary metals levels with further adjustment for survey cycle. **Table S5.** OR (95% CI) in OA associated with single and mixed urinary metals levels after excluding participants with abnormal urinary creatinine. **Table S6.** OR (95% CI) in OA associated with single and mixed urinary metals levels after excluding pregnant participants. **Table S7.** Biological aging markers as mediators in the associations of single metals with OA risk. **Table S8.** Telomere length and biological age/biological age as serial mediators in the associations of single metals with OA risk.

**Additional file 2. **Code for analyses.

## Data Availability

The National Health and Nutrition Examination Survey (NHANES) data are publicly available at https://www.cdc.gov/nchs/nhanes/index.htm.

## Abbreviations

Ba: Barium
Cd: Cadmium
CI: Confidence interval
Co: Cobalt
CRP: C-reactive protein
Cs: Cesium
ICP-MS: Inductively coupled plasma mass spectrometry
Mo: Molybdenum
NCHS: National Center for Health Statistics
NHANES: National Health and Nutrition Examination Survey
OA: Osteoarthritis
OR: Odds ratios
Pb: Lead
qPCR: Uantitative polymerase chain reaction
SASP: Senescence-related secretory phenotype
Sb: Antimony
SE: Standard error
Tl: Thallium
Tu: Uranium
WQS: Weighted quantile sum
