# Supplementary material for: Biological aging mediates the associations between urinary metals and osteoarthritis among U.S. adults
Source: BMC Med. 2022 Jun 17;20:207. doi: 10.1186/s12916-022-02403-3 (PMC9205020; doi:10.1186/s12916-022-02403-3)
Supplement: Supplementary file 1 — Additional file 1: Fig. S1. Directed acyclic graph. Fig. S2. Pearson’s correlation matrix. Fig. S3. Weighted values of urinary metals for OA in WQS models. Fig. S4. Associations of the urinary metals with OA risk estimated by Bayesian Kernel Machine Regression (BKMR). Table S1. Distributions of metals in the study population. Table S2. OR (95% CI) in OA associated with single and mixed urinary metals levels with further adjustment for occupation. Table S3. OR (95% CI) in OA associated with single and mixed urinary metals levels with further adjustment for other diseases and medicine use. Table S4. OR (95% CI) in OA associated with single and mixed urinary metals levels with further adjustment for survey cycle. Table S5. OR (95% CI) in OA associated with single and mixed urinary metals levels after excluding participants with abnormal urinary creatinine. Table S6. OR (95% CI) in OA associated with single and mixed urinary metals levels after excluding pregnant participants. Table S7. Biological aging markers as mediators in the associations of single metals with OA risk. Table S8. Telomere length and biological age/biological age as serial mediators in the associations of single metals with OA risk. [file 12916_2022_2403_MOESM1_ESM.doc]

**Additional file 1**

| Table S1. Distributions of metals in the study population. | | | |
| --- | --- | --- | --- |
| Metals (μg/L) | Detection rate (%) | Median | Interquartile range |
| Ba | 98.55 | 1.36 | 0.77-2.46 |
| Cd | 93.42 | 0.22 | 0.12-0.40 |
| Co | 99.63 | 0.33 | 0.23-0.51 |
| Cs | 99.99 | 4.36 | 3.23-6.06 |
| Mo | 99.95 | 38.70 | 26.45-56.95 |
| Pb | 95.56 | 0.48 | 0.29-0.79 |
| Sb | 76.56 | 0.06 | 0.04-0.10 |
| Tl | 99.32 | 0.16 | 0.11-0.22 |
| Tu | 90.15 | 0.07 | 0.04-0.12 |


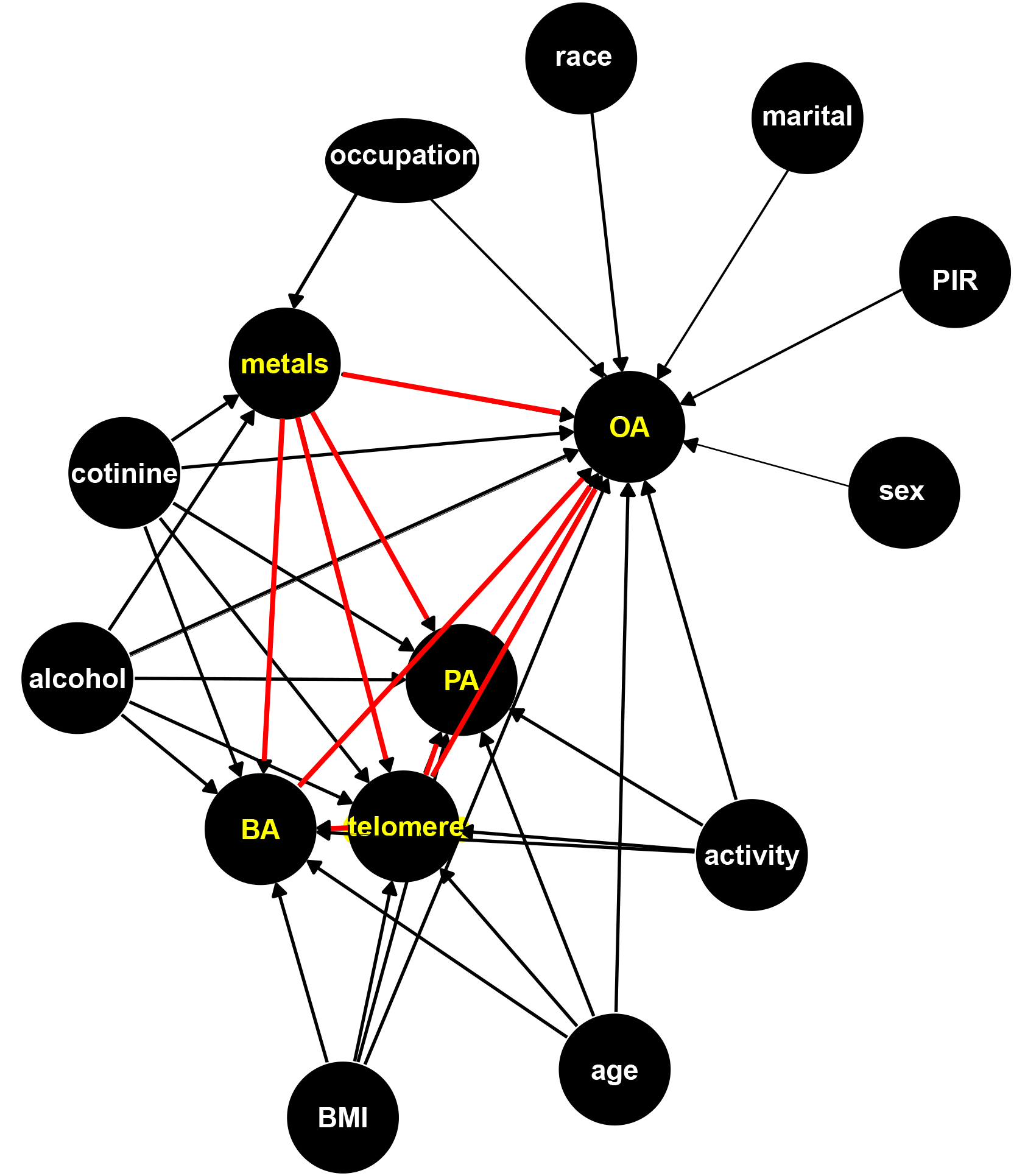


Fig. S1. Directed acyclic graph. The red lines showed the main paths of this analysis. OA, osteoarthritis; cotinine, serum cotinine concentration; activity, physical activity; alcohol, drinking alcohol status; BMI, body mass index; PIR, the ratio of family income to poverty; BA, Biological Age; PA, phenotypic Age; telomere, telomere length.


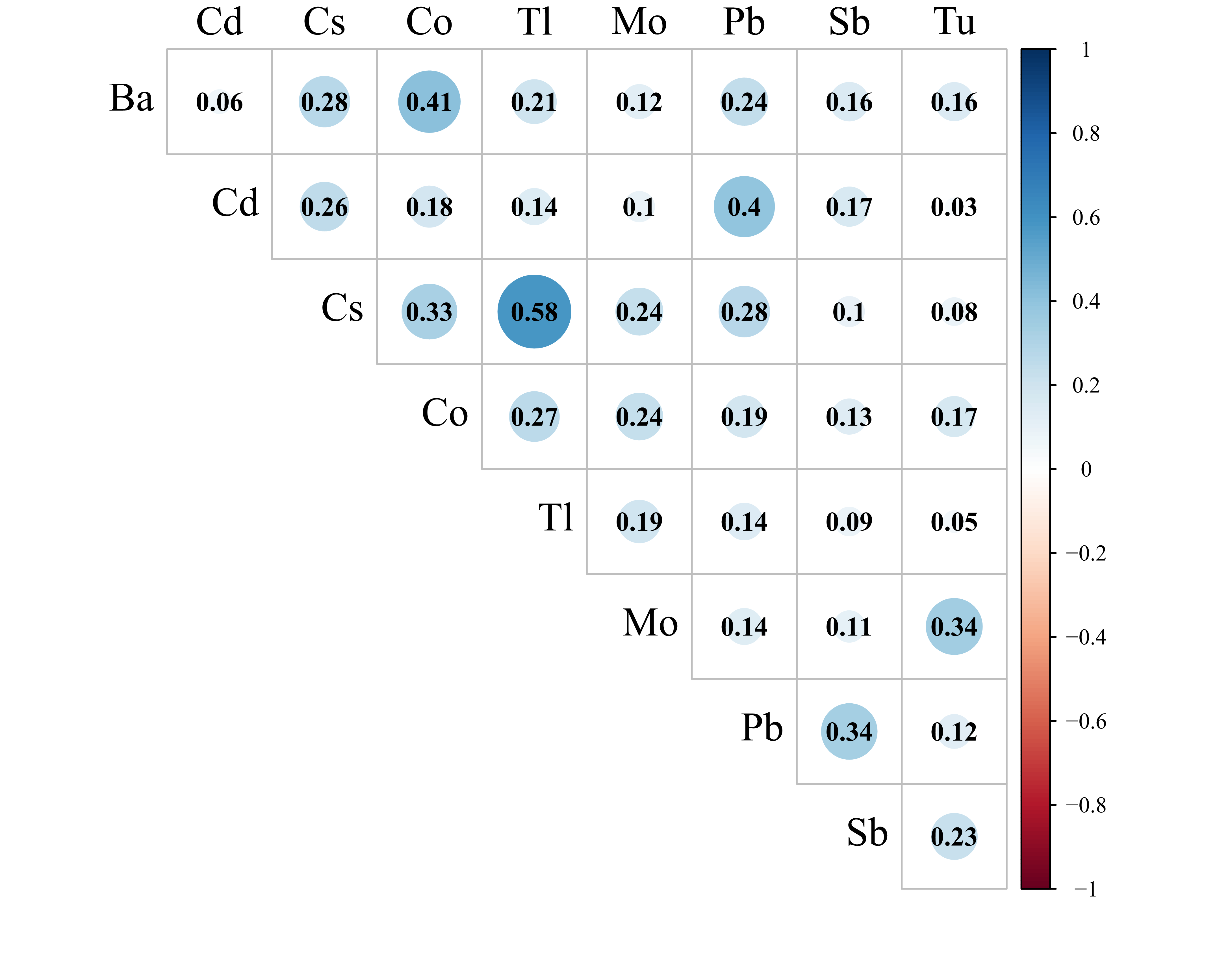


Fig. S2. Pearson’s correlation matrix among Ln-transformed urinary metals in the study population.


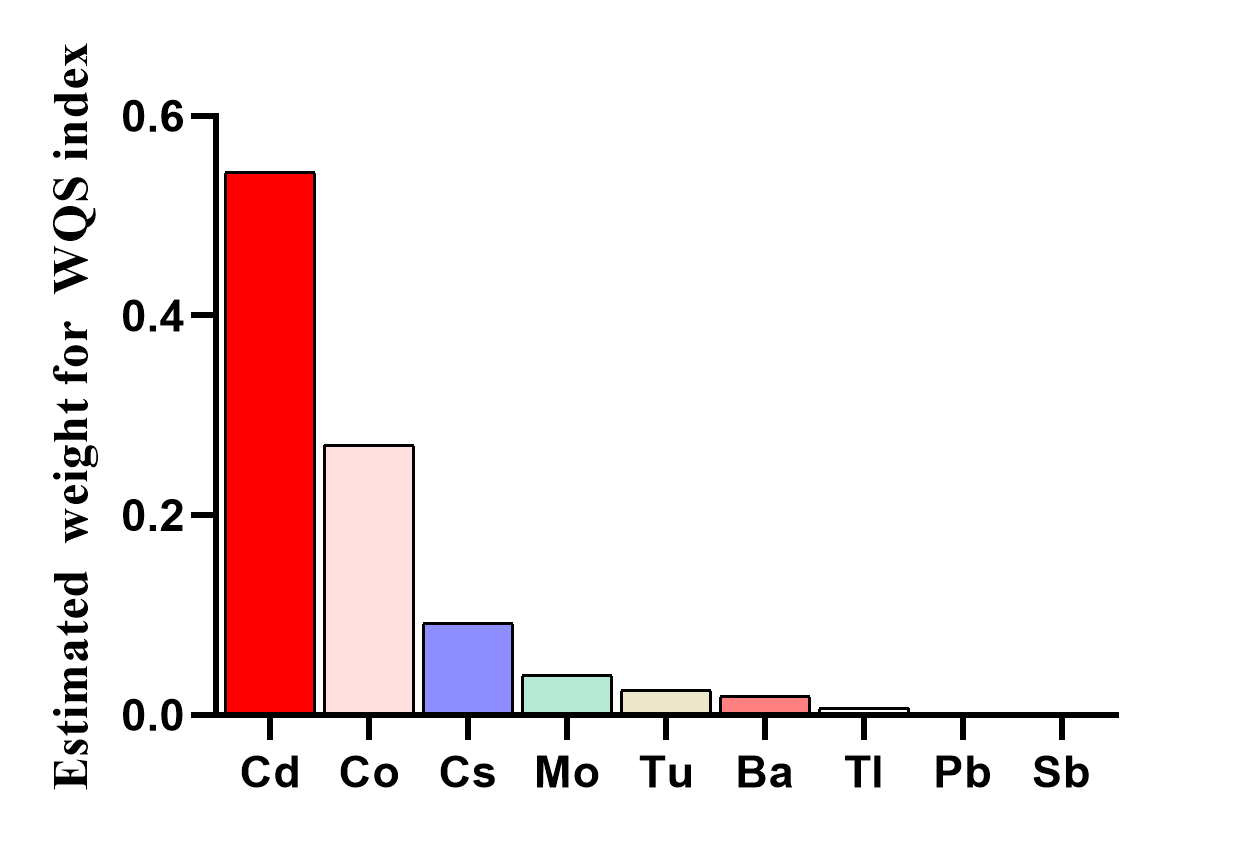


Fig. S3. Weighted values of urinary metals for OA in WQS models. Models were adjusted for sex, age, race/ethnicity, education, family income-to-poverty ratio, marital status, body mass index, physical activity, drinking alcohol status, and serum cotinine.

**
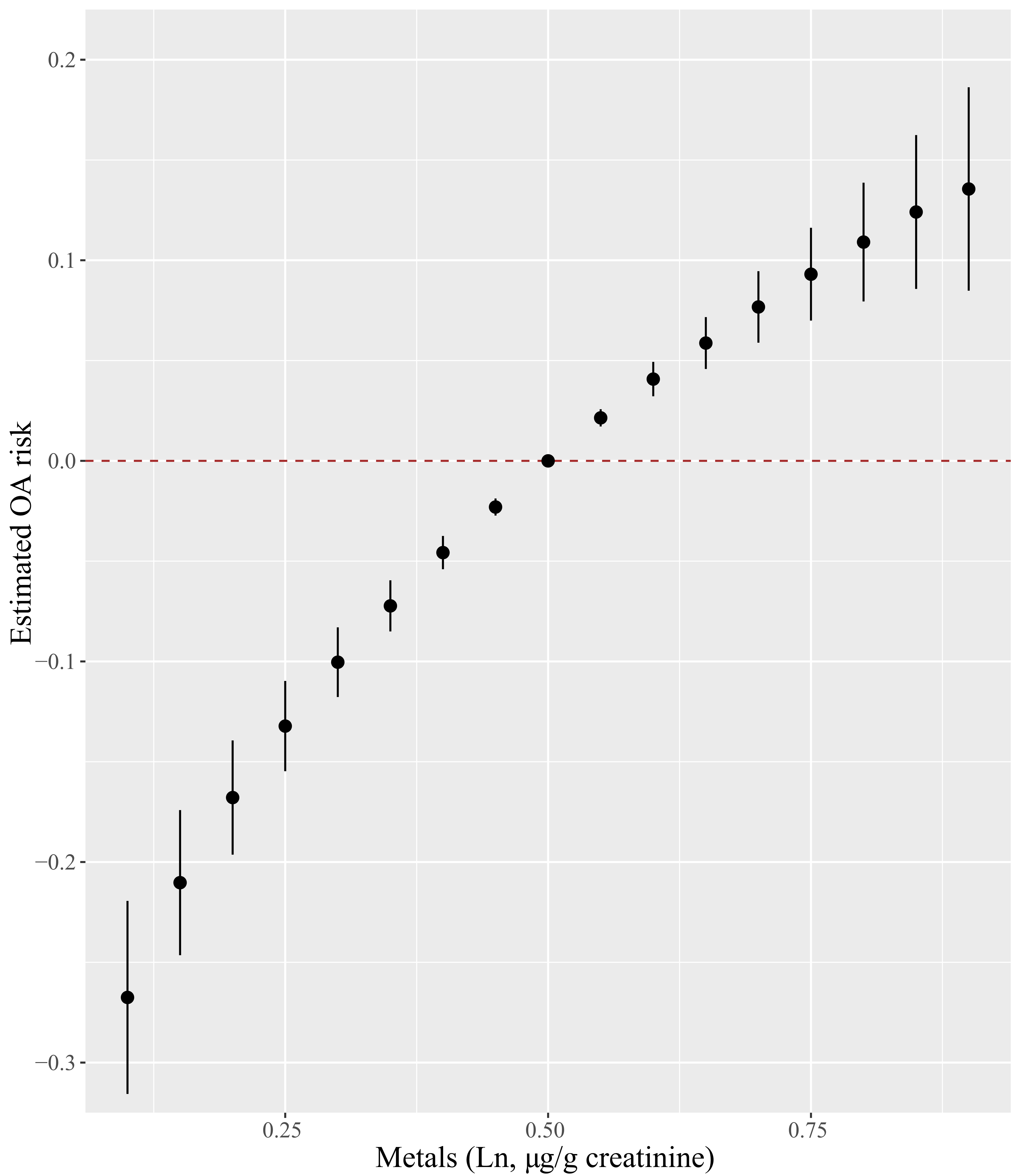

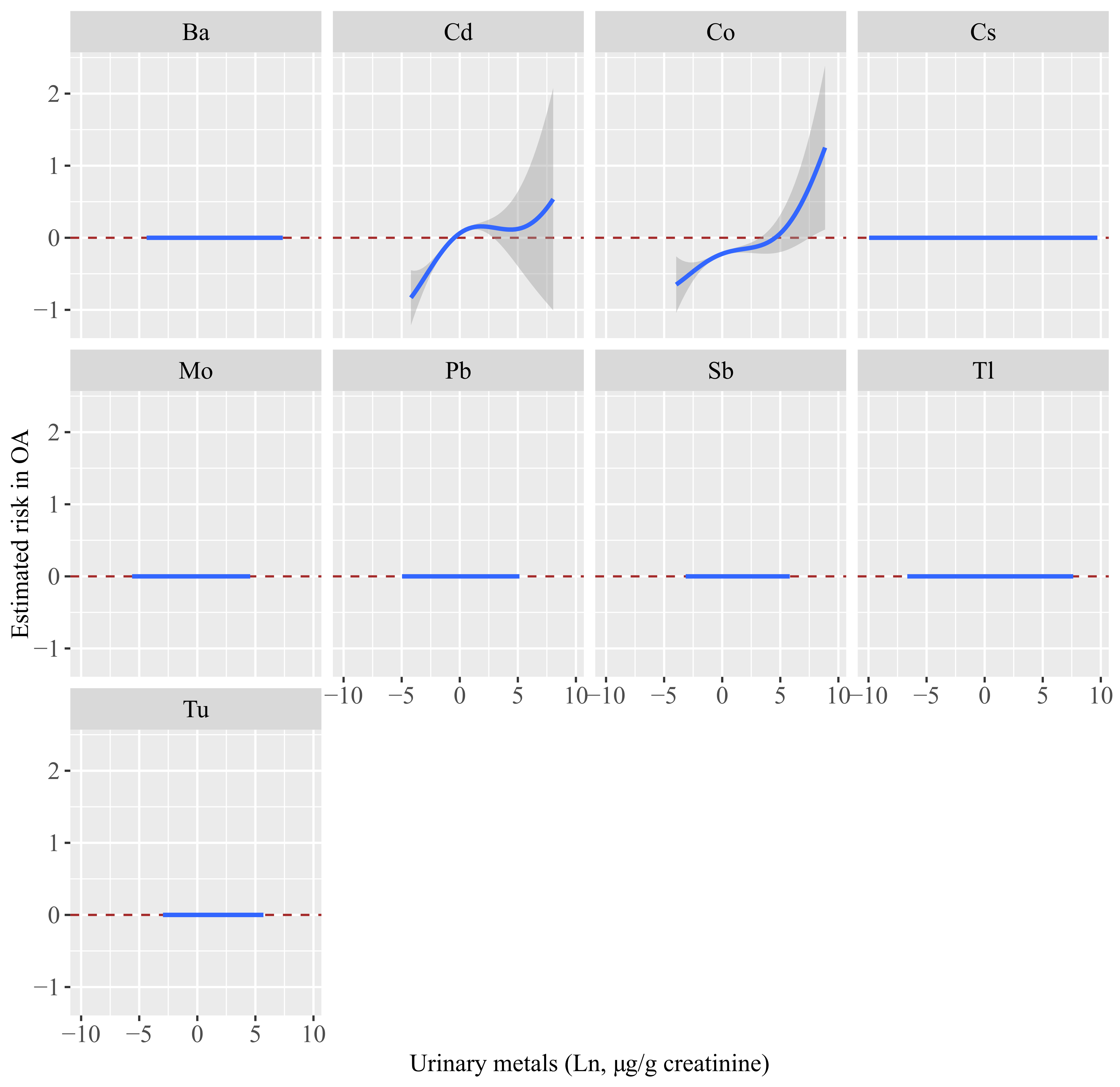
**

**A**

**B**

Fig. S4. Associations of the urinary metals with OA risk estimated by Bayesian Kernel Machine Regression (BKMR). (A) Exposure-response functions for each metals with the other metals fixed at the median. (B) Combined effects of urinary metals mixture on OA risk. This plot showed the estimated difference in OA risk and 95% confidence interval when all metals concentrations were held at particular percentiles compared to their medians. Models were adjusted for sex, age, race/ethnicity, education, family income-to-poverty ratio, marital status, body mass index, physical activity, drinking alcohol status, and serum cotinine.

| Table S2. OR (95% CI) in OA associated with single and mixed urinary metals levels with further adjustment for occupation | | | | | | |
| --- | --- | --- | --- | --- | --- | --- |
| Metals | Continuous | Q1 | Q2 | Q3 | Q4 | *P* for trend |
| (μg/g creatinine) | OR (95% CI) |  | OR (95% CI) | OR (95% CI) | OR (95% CI) |  |
| Ba | 1.01 (0.93, 1.10) | 1.00 (reference) | 0.91 (0.72, 1.15) | 0.91 (0.73, 1.13) | 0.98 (0.79, 1.22) | 0.994 |
| Cd | 1.24 (1.10, 1.39) | 1.00 (reference) | 1.67 (1.24, 2.25) | 1.77 (1.29, 2.44) | 1.69 (1.24, 2.30) | 0.010 |
| Co | 1.26 (1.12, 1.42) | 1.00 (reference) | 1.27 (0.97, 1.66) | 1.42 (1.09, 1.86) | 1.59 (1.21, 2.09) | 0.005 |
| Cs | 1.39 (1.17, 1.65) | 1.00 (reference) | 1.12 (0.89, 1.42) | 1.27 (0.99, 1.63) | 1.47 (1.13, 1.92) | 0.007 |
| Mo | 0.95 (0.85, 1.07) | 1.00 (reference) | 1.16 (0.95, 1.41) | 1.18 (0.93, 1.50) | 1.00 (0.80, 1.26) | 0.994 |
| Pb | 1.09 (0.96, 1.23) | 1.00 (reference) | 1.24 (0.96, 1.61) | 1.04 (0.80, 1.36) | 1.15 (0.88, 1.49) | 0.930 |
| Sb | 0.93 (0.83, 1.04) | 1.00 (reference) | 1.00 (0.79, 1.28) | 0.97 (0.77, 1.23) | 0.86 (0.67, 1.10) | 0.390 |
| Tl | 0.98 (0.82, 1.17) | 1.00 (reference) | 0.92 (0.72, 1.17) | 0.79 (0.63, 1.00) | 0.99 (0.77, 1.27) | 0.994 |
| Tu | 1.01 (0.93, 1.10) | 1.00 (reference) | 1.04 (0.83, 1.31) | 1.10 (0.88, 1.37) | 1.07 (0.86, 1.32) | 0.802 |
| Mixed metals | 1.24 (1.11, 1.39) | 1.00 (reference) | 1.26 (1.01, 1.56) | 1.28 (1.04, 1.58) | 1.55 (1.26, 1.92) | <0.001 |
| Models were adjusted for sex, age, race/ethnicity, education, family income-to-poverty ratio, marital status, body mass index, physical activity, drinking alcohol status, serum cotinine, and occupation. Continuous, Ln-transformed concentration of metals; Q, quartile; OA, osteoarthritis. All *P* for trend were FDR-adjusted. | | | | | | |

| Table S3. OR (95% CI) in OA associated with single and mixed urinary metals levels with further adjustment for other diseases and medicine use | | | | | | |
| --- | --- | --- | --- | --- | --- | --- |
| Metals | Continuous | Q1 | Q2 | Q3 | Q4 | *P* for trend |
| (μg/g creatinine) | OR (95% CI) |  | OR (95% CI) | OR (95% CI) | OR (95% CI) |  |
| Ba | 1.03 (0.94, 1.12) | 1.00 (reference) | 0.92 (0.73, 1.16) | 0.92 (0.74, 1.15) | 1.02 (0.82, 1.27) | 0.811 |
| Cd | 1.31 (1.17, 1.47) | 1.00 (reference) | 1.76 (1.31, 2.37) | 1.94 (1.41, 2.67) | 1.94 (1.42, 2.63) | 0.001 |
| Co | 1.22 (1.09, 1.38) | 1.00 (reference) | 1.23 (0.94, 1.61) | 1.34 (1.02, 1.76) | 1.48 (1.12, 1.94) | 0.018 |
| Cs | 1.41 (1.19, 1.68) | 1.00 (reference) | 1.15 (0.91, 1.45) | 1.33 (1.03, 1.70) | 1.52 (1.16, 1.98) | 0.003 |
| Mo | 0.98 (0.88, 1.10) | 1.00 (reference) | 1.19 (0.97, 1.45) | 1.22 (0.97, 1.55) | 1.05 (0.83, 1.33) | 0.811 |
| Pb | 1.22 (1.07, 1.39) | 1.00 (reference) | 1.38 (1.06, 1.81) | 1.24 (0.94, 1.64) | 1.47 (1.10, 1.97) | 0.062 |
| Sb | 1.01 (0.89, 1.14) | 1.00 (reference) | 1.06 (0.83, 1.36) | 1.07 (0.84, 1.36) | 1.01 (0.78, 1.30) | 0.953 |
| Tl | 0.97 (0.81, 1.16) | 1.00 (reference) | 0.91 (0.71, 1.16) | 0.78 (0.62, 0.99) | 0.97 (0.75, 1.25) | 0.811 |
| Tu | 1.03 (0.95, 1.13) | 1.00 (reference) | 1.07 (0.85, 1.34) | 1.15 (0.92, 1.43) | 1.13 (0.91, 1.39) | 0.362 |
| Mixed metals | 1.26 (1.13, 1.39) | 1.00 (reference) | 1.36 (1.09, 1.70) | 1.52 (1.23, 1.87) | 1.68 (1.36, 2.09) | <0.001 |
| Models were adjusted for sex, age, race/ethnicity, education, family income-to-poverty ratio, marital status, body mass index, physical activity, drinking alcohol status, serum cotinine, disease, and medicine use. Continuous, Ln-transformed concentration of metals; Q, quartile; OA, osteoarthritis. All *P* for trend were FDR-adjusted. | | | | | | |

| Table S4. OR (95% CI) in OA associated with single and mixed urinary metals levels with further adjustment for survey cycle | | | | | | |
| --- | --- | --- | --- | --- | --- | --- |
| Metals | Continuous | Q1 | Q2 | Q3 | Q4 | *P* for trend |
|  | OR (95% CI) |  | OR (95% CI) | OR (95% CI) | OR (95% CI) |  |
| Ba | 1.03 (0.94, 1.12) | 1.00 (reference) | 0.91 (0.72, 1.15) | 0.92 (0.73, 1.15) | 1.01 (0.81, 1.27) | 0.760 |
| Cd | 1.33 (1.18, 1.49) | 1.00 (reference) | 1.78 (1.32, 2.40) | 2.00 (1.46, 2.75) | 2.02 (1.48, 2.75) | <0.001 |
| Co | 1.22 (1.08, 1.38) | 1.00 (reference) | 1.24 (0.95, 1.61) | 1.34 (1.03, 1.75) | 1.48 (1.13, 1.93) | 0.018 |
| Cs | 1.39 (1.17, 1.66) | 1.00 (reference) | 1.14 (0.90, 1.44) | 1.32 (1.03, 1.70) | 1.49 (1.14, 1.94) | 0.007 |
| Mo | 0.98 (0.88, 1.10) | 1.00 (reference) | 1.19 (0.98, 1.46) | 1.21 (0.96, 1.54) | 1.05 (0.84, 1.32) | 0.760 |
| Pb | 1.26 (1.11, 1.43) | 1.00 (reference) | 1.40 (1.08, 1.83) | 1.28 (0.97, 1.68) | 1.57 (1.18, 2.08) | 0.020 |
| Sb | 1.06 (0.94, 1.21) | 1.00 (reference) | 1.07 (0.84, 1.37) | 1.12 (0.88, 1.43) | 1.12 (0.86, 1.46) | 0.510 |
| Tl | 0.97 (0.81, 1.16) | 1.00 (reference) | 0.93 (0.73, 1.19) | 0.80 (0.63, 1.01) | 0.99 (0.76, 1.27) | 0.760 |
| Tu | 1.03 (0.95, 1.12) | 1.00 (reference) | 1.08 (0.86, 1.36) | 1.16 (0.93, 1.44) | 1.12 (0.91, 1.38) | 0.393 |
| Mixed metals | 1.25 (1.14, 1.39) | 1.00 (reference) | 1.33 (1.07, 1.66) | 1.53 (1.24, 1.89) | 1.66 (1.34, 2.07) | <0.001 |
| Models were adjusted for sex, age, race/ethnicity, education, family income-to-poverty ratio, marital status, body mass index, physical activity, drinking alcohol status, and serum cotinine. Continuous, Ln-transformed concentration of metals; Q, quartile; OA, osteoarthritis. All *P* for trend were FDR-adjusted. | | | | | | |

| Table S5. OR (95% CI) in OA associated with single and mixed urinary metals levels after excluding participants with abnormal urinary creatinine | | | | | | |
| --- | --- | --- | --- | --- | --- | --- |
| Metals | Continuous | Q1 | Q2 | Q3 | Q4 | *P* for trend |
| (μg/g creatinine) | OR (95% CI) |  | OR (95% CI) | OR (95% CI) | OR (95% CI) |  |
| Ba | 1.00 (0.92, 1.09) | 1.00 (reference) | 0.92 (0.72, 1.17) | 0.87 (0.70, 1.09) | 0.93 (0.74, 1.17) | 0.799 |
| Cd | 1.22 (1.07, 1.38) | 1.00 (reference) | 1.55 (1.13, 2.13) | 1.68 (1.20, 2.35) | 1.56 (1.13, 2.15) | 0.045 |
| Co | 1.29 (1.13, 1.48) | 1.00 (reference) | 1.20 (0.91, 1.58) | 1.46 (1.12, 1.90) | 1.59 (1.20, 2.11) | 0.010 |
| Cs | 1.32 (1.09, 1.61) | 1.00 (reference) | 1.15 (0.89, 1.49) | 1.27 (0.98, 1.64) | 1.40 (1.05, 1.88) | 0.045 |
| Mo | 0.93 (0.82, 1.06) | 1.00 (reference) | 1.08 (0.89, 1.31) | 1.11 (0.87, 1.42) | 0.95 (0.74, 1.21) | 0.839 |
| Pb | 1.07 (0.93, 1.23) | 1.00 (reference) | 1.18 (0.90, 1.55) | 0.94 (0.71, 1.25) | 1.12 (0.84, 1.49) | 0.850 |
| Sb | 0.93 (0.82, 1.06) | 1.00 (reference) | 0.99 (0.80, 1.24) | 0.91 (0.70, 1.18) | 0.84 (0.66, 1.07) | 0.238 |
| Tl | 0.88 (0.73, 1.07) | 1.00 (reference) | 0.91 (0.70, 1.18) | 0.82 (0.65, 1.05) | 0.85 (0.65, 1.10) | 0.275 |
| Tu | 1.01 (0.92, 1.10) | 1.00 (reference) | 1.03 (0.82, 1.29) | 0.98 (0.78, 1.23) | 1.05 (0.85, 1.30) | 0.839 |
| Mixed metals | 1.19 (1.03, 1.39) | 1.00 (reference) | 1.09 (0.89, 1.35) | 1.15 (0.93, 1.42) | 1.30 (1.05, 1.61) | 0.045 |
| Models were adjusted for sex, age, race/ethnicity, education, family income-to-poverty ratio, marital status, body mass index, physical activity, drinking alcohol status, and serum cotinine. Continuous, Ln-transformed concentration of metals; Q, quartile; OA, osteoarthritis. All *P* for trend were FDR-adjusted. | | | | | | |

| Table S6. OR (95% CI) in OA associated with single and mixed urinary metals levels after excluding pregnant participants | | | | | | |
| --- | --- | --- | --- | --- | --- | --- |
| Metals | Continuous | Q1 | Q2 | Q3 | Q4 | *P* for trend |
| (μg/g creatinine) | OR (95% CI) |  | OR (95% CI) | OR (95% CI) | OR (95% CI) |  |
| Ba | 1.01 (0.93, 1.09) | 1.00 (reference) | 0.90 (0.71, 1.14) | 0.86 (0.69, 1.06) | 0.97 (0.78, 1.21) | 0.897 |
| Cd | 1.25 (1.12, 1.41) | 1.00 (reference) | 1.58 (1.17, 2.15) | 1.74 (1.26, 2.41) | 1.70 (1.25, 2.33) | 0.010 |
| Co | 1.25 (1.10, 1.41) | 1.00 (reference) | 1.28 (0.98, 1.66) | 1.40 (1.07, 1.83) | 1.55 (1.17, 2.06) | 0.010 |
| Cs | 1.33 (1.12, 1.58) | 1.00 (reference) | 1.10 (0.86, 1.40) | 1.23 (0.95, 1.59) | 1.41 (1.08, 1.85) | 0.020 |
| Mo | 0.94 (0.84, 1.06) | 1.00 (reference) | 1.11 (0.91, 1.35) | 1.15 (0.90, 1.46) | 0.97 (0.77, 1.22) | 0.897 |
| Pb | 1.07 (0.94, 1.22) | 1.00 (reference) | 1.18 (0.91, 1.53) | 1.01 (0.77, 1.32) | 1.11 (0.85, 1.44) | 0.897 |
| Sb | 0.93 (0.82, 1.04) | 1.00 (reference) | 1.01 (0.79, 1.28) | 0.95 (0.74, 1.20) | 0.86 (0.67, 1.10) | 0.350 |
| Tl | 0.93 (0.78, 1.12) | 1.00 (reference) | 0.89 (0.70, 1.14) | 0.77 (0.61, 0.98) | 0.93 (0.72, 1.19) | 0.723 |
| Tu | 1.00 (0.92, 1.09) | 1.00 (reference) | 1.00 (0.80, 1.26) | 1.08 (0.87, 1.35) | 1.01 (0.82, 1.25) | 0.897 |
| Mixed metals | 1.29 (1.12, 1.48) | 1.00 (reference) | 1.37 (1.11, 1.70) | 1.28 (1.03, 1.58) | 1.56 (1.26, 1.94) | <0.001 |
| Models were adjusted for sex, age, race/ethnicity, education, family income-to-poverty ratio, marital status, body mass index, physical activity, drinking alcohol status, and serum cotinine. Continuous, Ln-transformed concentration of metals; Q, quartile; OA, osteoarthritis. All *P* for trend were FDR-adjusted. | | | | | | |

| Table S7. Biological aging markers as mediators in the associations of single metals with OA risk | | | | | | |
| --- | --- | --- | --- | --- | --- | --- |
| Metals | Biological Age | *P* value | Phenotypic Age | *P* value | Telomere length | *P* value |
|  | Estimation% (95% CI) |  | Estimation% (95% CI) |  | Estimation% (95% CI) |  |
| Ba |  |  |  |  |  |  |
| Total effect | 0.03 (-0.10, 0.10) | 0.260 | 0.04 (-0.04, 0.17) | 0.260 | 0.81 (-0.21, 2.00) | 0.140 |
| Indirect effect | 0.00 (0.00, 0.03) | 0.880 | 0.01 (-0.01, 0.01) | 0.280 | 0.04 (-0.02, 0.12) | 0.240 |
| Direct effect | 0.03 (-0.11, 0.1) | 0.280 | 0.03 (-0.04, 0.17) | 0.280 | 0.77 (-0.23, 1.96) | 0.200 |
| Cd |  |  |  |  |  |  |
| Total effect | 4.23 (2.79, 6.09) | <0.001 | 3.54 (2.29, 5.30) | <0.001 | 1.40 (-0.34, 4.62) | 0.240 |
| Indirect effect | 2.93 (2.45, 3.75) | <0.001 | 2.16 (1.67, 2.76) | <0.001 | 0.34 (0.05, 0.75) | 0.020 |
| Direct effect | 1.29 (-0.04, 2.70) | 0.080 | 1.38 (0.45, 2.91) | 0.040 | 1.05 (-0.64, 4.13) | 0.380 |
| Co |  |  |  |  |  |  |
| Total effect | 2.55 (1.34, 4.12) | <0.001 | 2.45 (1.11, 3.51) | <0.001 | 0.36 (-1.33, 2.12) | 0.520 |
| Indirect effect | 0.47 (0.27, 0.67) | <0.001 | 0.78 (0.58, 1.01) | <0.001 | 0.06 (-0.04, 0.21) | 0.240 |
| Direct effect | 2.08 (0.92, 3.62) | <0.001 | 1.68 (0.35, 2.83) | <0.001 | 0.30 (-1.36, 2.05) | 0.500 |
| Cs |  |  |  |  |  |  |
| Total effect | 2.59 (1.92, 3.07) | <0.001 | 2.47 (1.76, 3.02) | <0.001 | 2.14 (0.67, 2.87) | 0.040 |
| Indirect effect | 0.79 (0.58, 0.98) | <0.001 | 0.42 (0.27, 0.55) | <0.001 | 0.21 (0.05, 0.56) | <0.001 |
| Direct effect | 1.80 (1.08, 2.46) | <0.001 | 2.06 (1.36, 2.63) | <0.001 | 1.94 (0.38, 2.58) | 0.040 |
| Mo |  |  |  |  |  |  |
| Total effect | 0.14 (-0.69, 0.73) | 0.640 | 0.10 (-0.79, 0.78) | 0.980 | 0.38 (-1.87, 0.98) | 0.600 |
| Indirect effect | 0.13 (-0.01, 0.31) | 0.060 | 0.00 (-0.12, 0.13) | 0.900 | 0.05 (-0.05, 0.19) | 0.240 |
| Direct effect | 0.01 (-0.84, 0.64) | 0.860 | 0.10 (-0.79, 0.80) | 0.980 | 0.33 (-2.04, 0.92) | 0.660 |
| Pb |  |  |  |  |  |  |
| Total effect | 1.18 (0.22, 2.49) | 0.040 | 0.99 (-0.13, 2.35) | 0.060 | 0.06 (-1.48, 1.88) | 0.920 |
| Indirect effect | 1.38 (1.05, 1.68) | <0.001 | 1.00 (0.78, 1.26) | <0.001 | 0.01 (-0.17, 0.17) | 0.940 |
| Direct effect | -0.19 (-1.15, 1.04) | 0.640 | -0.01 (-1.02, 1.20) | 0.960 | 0.05 (-1.49, 1.91) | 0.900 |
| Sb |  |  |  |  |  |  |
| Total effect | -1.71 (-6.36, 3.83) | 0.580 | -1.93 (-5.89, 3.09) | 0.460 | -1.47 (-7.58, 4.00) | 0.440 |
| Indirect effect | 1.05 (0.41, 2.06) | <0.001 | 0.62 (0.04, 1.21) | 0.060 | -0.07 (-0.52, 0.58) | 0.720 |
| Direct effect | -2.76 (-7.09, 2.36) | 0.300 | -2.55 (-6.44, 2.01) | 0.280 | -1.40 (-7.54, 3.73) | 0.440 |
| Tl |  |  |  |  |  |  |
| Total effect | -1.68 (-5.37, 3.20) | 0.680 | -1.91 (-5.7, 2.51) | 0.380 | -0.53 (-1.00, 1.07) | 0.460 |
| Indirect effect | 1.03 (0.38, 1.67) | <0.001 | 0.63 (0.09, 1.48) | 0.040 | 0.05 (-0.03, 0.19) | 0.140 |
| Direct effect | -2.71 (-6.29, 2.18) | 0.260 | -2.53 (-6.36, 1.76) | 0.260 | -0.58 (-1.02, 0.88) | 0.380 |
| Tu |  |  |  |  |  |  |
| Total effect | 0.90 (-0.01, 1.86) | 0.060 | 0.85 (0.07, 1.70) | 0.020 | 1.17 (-0.81, 6.05) | 0.420 |
| Indirect effect | 0.07 (-0.05, 0.18) | 0.300 | 0.06 (-0.04, 0.17) | 0.240 | 0.11 (-0.08, 0.55) | 0.340 |
| Direct effect | 0.83 (-0.06, 1.81) | 0.080 | 0.79 (0.04, 1.65) | 0.040 | 1.06 (-0.88, 5.82) | 0.480 |
| Models were adjusted for sex, age, race/ethnicity, education, family income-to-poverty ratio, marital status, physical activity, drinking alcohol status, serum cotinine, and body mass index. | | | | | | |

| Table S8. Telomere length and Biological Age/Biological Age as serial mediators in the associations of single metals with OA risk | | | | |
| --- | --- | --- | --- | --- |
| Metals | Telomere length-Biological Age | | Telomere length-Phenotypic Age | |
|  | Estimation% (95% CI) | *P* value | Estimation% (95% CI) | *P* value |
| Ba |  |  |  |  |
| Total effect | 0.80 (-0.20, 2.00) | 0.195 | 0.80 (-0.50, 2.00) | 0.198 |
| Serial mediated effect | 0.00 (0.00, 0.00) | 0.373 | 0.00 (0.00, 0.00) | 0.370 |
| Direct effect | 0.80 (-0.10, 2.20) | 0.161 | 0.80 (-0.40, 2.10) | 0.186 |
| Cd |  |  |  |  |
| Total effect | 0.60 (-0.80, 2.10) | 0.455 | 0.60 (-0.90, 2.00) | 0.446 |
| Serial mediated effect | 0.10 (0.00, 0.20) | 0.002 | 0.10 (0.00, 0.10) | 0.009 |
| Direct effect | -1.30 (-2.90, 0.50) | 0.116 | -1.00 (-2.70, 0.60) | 0.209 |
| Co |  |  |  |  |
| Total effect | -0.40 (-2.40, 1.70) | 0.690 | -0.20 (-2.00, 1.80) | 0.813 |
| Serial mediated effect | 0.00 (0.00, 0.10) | 0.542 | 0.00 (0.00, 0.10) | 0.456 |
| Direct effect | -0.70 (-2.60, 1.40) | 0.497 | -0.90 (-2.90, 1.30) | 0.385 |
| Cs |  |  |  |  |
| Total effect | 2.20 (0.10, 4.40) | 0.047 | 2.40 (0.20, 4.60) | 0.031 |
| Serial mediated effect | 0.10 (0.00, 0.20) | 0.010 | 0.10 (0.00, 0.10) | 0.025 |
| Direct effect | 1.20 (-1.00, 3.30) | 0.293 | 1.60 (-0.60, 3.70) | 0.152 |
| Mo |  |  |  |  |
| Total effect | 0.30 (-1.60, 1.80) | 0.733 | 0.50 (-0.90, 2.20) | 0.570 |
| Serial mediated effect | 0.00 (0.00, 0.10) | 0.170 | 0.00 (0.00, 0.10) | 0.459 |
| Direct effect | 0.10 (-1.70, 1.50) | 0.910 | 0.40 (-1.20, 2.10) | 0.631 |
| Pb |  |  |  |  |
| Total effect | 0.00 (-1.80, 1.70) | 0.982 | 0.10 (-1.60, 1.70) | 0.918 |
| Serial mediated effect | 0.00 (0.00, 0.00) | 0.917 | 0.00 (0.00, 0.00) | 0.918 |
| Direct effect | -0.80 (-2.70, 1.00) | 0.398 | -0.60 (-2.40, 1.00) | 0.543 |
| Sb |  |  |  |  |
| Total effect | -1.7 (-6.8, 4.6) | 0.537 | -1.6 (-6.6, 4) | 0.594 |
| Serial mediated effect | 0 (-0.2, 0.2) | 0.724 | 0 (-0.2, 0.1) | 0.765 |
| Direct effect | -2.3 (-8.3, 3.4) | 0.401 | -2.1 (-7.3, 3.6) | 0.484 |
| Tl |  |  |  |  |
| Total effect | -0.60 (-1.90, 0.60) | 0.341 | -0.60 (-1.80, 0.50) | 0.363 |
| Serial mediated effect | 0.00 (0.00, 0.00) | 0.176 | 0.00 (0.00, 0.00) | 0.222 |
| Direct effect | -0.70 (-2.00, 0.60) | 0.271 | -0.60 (-1.90, 0.40) | 0.309 |
| Tu |  |  |  |  |
| Total effect | 1.10 (-1.00, 3.60) | 0.424 | 1.00 (-1.00, 3.20) | 0.424 |
| Serial mediated effect | 0.00 (0.00, 0.10) | 0.401 | 0.00 (0.00, 0.10) | 0.364 |
| Direct effect | 0.60 (-1.60, 3.10) | 0.676 | 0.80 (-1.20, 3.00) | 0.533 |
| Models were adjusted for sex, age, race/ethnicity, education, family income-to-poverty ratio, marital status, physical activity, drinking alcohol status, serum cotinine, and body mass index. | | | | |
