# Supplementary material for: Biological aging mediates the associations between urinary metals and osteoarthritis among U.S. adults
Source: BMC Med. 2022 Jun 17;20:207. doi: 10.1186/s12916-022-02403-3 (PMC9205020; doi:10.1186/s12916-022-02403-3)
Supplement: Supplementary file 2 — Additional file 2. Code for analyses. [file 12916_2022_2403_MOESM2_ESM.docx]

**Additional file 2**

**(****code for analyses)**

**1.Multivariable logistic regression (SAS)**

**proc** **surveylogistic** data=a;

STRATA sdmvstra;CLUSTER sdmvpsu;WEIGHT wtmec20yr;

class RIAGENDR(param=ref ref="1") DMDEDUC2(param=ref ref="1") DMDMARTL(param=ref ref="1") alq101(param=ref ref="1") RIDRETH1(param=ref ref="1") phy(param=ref ref="1") age(param=ref ref="1");

model OA(event="1")=Cd RIAGENDR age RIDRETH1 DMDEDUC2 DMDMARTL alq101 lbxcot INDFMPIR phy bmxbmi/vadjust=none;

**run**;

**2.WQS (R)**

rm(list = ls())

library(gWQS)

library(ggplot2)

library(epiDisplay)

data = read.csv(file = "C:\\Users\\Administrator\\Desktop\\wqs.csv")

name <- names(data)[139:147]

data$age=as.factor(data$age)

data$RIAGENDR=as.factor(data$RIAGENDR)

data$RIDRETH1=as.factor(data$RIDRETH1)

data$DMDMARTL=as.factor(data$DMDMARTL)

data$DMDEDUC2=as.factor(data$DMDEDUC2)

data$phy=as.factor(data$phy)

data$alq101=as.factor(data$alq101)

results1=gwqs(OA ~ wqs +age +RIAGENDR +RIDRETH1 +DMDEDUC2 +INDFMPIR +BMXBMI +alq101+DMDMARTL+ LBXCOT+phy, mix_name = name, data = data, q = 4, validation = 0.6,b = 1000, b1_pos = TRUE, b1_constr = FALSE, family = "binomial", seed = 1003)

summary(results1)

gwqs_barplot(results1)

gwqs_scatterplot(results1)

gwqs_fitted_vs_resid(results1)

ptbp<-results1$final_weights

round(ptbp$mean_weight,4)

gwqs_summary_tab(results1)

ptbp

OR_ptb<-logistic.display(results1$fit)

OR_ptb

**3.BKMR (R)**

rm(list = ls())

library(bkmr)

library(ggplot2)

data1<-read.csv("C:\\Users\\Administrator\\Desktop\\bkmr.csv")

covar <- data.matrix(data1[, c("age", "RIAGENDR", "RIDRETH1" , "DMDEDUC2" , "DMDMARTL" , "INDFMPIR" , "BMXBMI" , "alq101" , "LBXCOT", "phy")])

expos <- data.matrix(data1[, c("Ba", "Cd", "Co","Cs", "Mo", "Pb","Sb", "Tl", "Tu")])

Y <- data1$OA

scale_expos <- scale(expos)

set.seed(1000)

knots50 <- fields::cover.design(scale_expos, nd = 50)$design

fitkm <- kmbayes(Y, Z = scale_expos, X = covar, iter = 10000, family = "binomial", est.h = TRUE, verbose = FALSE, varsel = TRUE,knots = knots50)

TracePlot(fit = fitkm, par = "beta")

TracePlot(fit = fitkm, par = "sigsq.eps")

TracePlot(fit = fitkm, par = "r", comp = 12)

ExtractPIPs(fitkm)

pred.resp.univar <- PredictorResponseUnivar(fit = fitkm,q.fixed=0.5)

ggplot(pred.resp.univar, aes(z, est, ymin = est - 1.96*se,

ymax = est + 1.96*se)) +

geom_hline(yintercept = 0, lty = 2, col = "brown")+

geom_smooth(stat = "identity") +

facet_wrap(~variable, ncol = 4) +

xlab("Urinary metals (Ln, ug/g creatinine)") +

ylab("Estimated risk in OA")+

theme(plot.title = element_text(hjust = 0.5,size = 12, family="serif"),axis.text=element_text(size=12,family="serif"),axis.title.x=element_text(size=12,family="serif"),axis.title.y=element_text(size=12,family="serif"),strip.text=element_text(size=12,color="black", family="serif"))+

theme(legend.title=element_text(size=12,family="serif"))

ggsave(filename="C:/Users/Administrator/Desktop/b1-1.tiff",plot=plot_1,width =5, height = 6)

library(eoffice)

graph2ppt(file="C:/Users/Administrator/Desktop/b1-1.tiff")

risks.overall <- OverallRiskSummaries(fit = fitkm, qs = seq(0.1, 0.9, by = 0.05), q.fixed = 0.5)

risks.overall

ggplot(risks.overall, aes(quantile, est, ymin = est - 1.96*sd,

ymax = est + 1.96*sd)) +

coord_cartesian(ylim = c(-0.3,0.2),xlim = c(0.1,0.9))+

geom_hline(yintercept = 0, lty = 2, col = "brown") +

geom_pointrange()+

xlab("Metals (Ln, ug/g creatinine)") +

ylab("Estimated OA risk")+

theme(plot.title = element_text(hjust = 0.5,size = 12, family="serif"),axis.text=element_text(size=12,family="serif"),axis.title.x=element_text(size=12,family="serif"),axis.title.y=element_text(size=12,family="serif"),strip.text=element_text(size=12,color="black", family="serif"))+

theme(legend.title=element_text(size=12,family="serif"))

**4.Parallel mediation (R)**

library(sandwich)

library(Hmisc)

library(MASS)

library(Matrix)

library(mvtnorm)

library(mediation)

rm(list = ls())

data = read.csv(file = "C:\\Users\\Administrator\\Desktop\\mediation.csv")

set.seed(2018)

M<-lm(ba~Cd+age+RIAGENDR+RIDRETH1+DMDEDUC2+INDFMPIR+BMXBMI+alq101+DMDMARTL+LBXCOT+phy, data=data)

Y<-glm(OA~ba+Cd+age+RIAGENDR+RIDRETH1+DMDEDUC2+INDFMPIR+BMXBMI+alq101+DMDMARTL+LBXCOT+phy, family="binomial", data=data)

results<-mediate(M,Y,treat='Cd',mediator='ba',boot=TRUE,sims=1000)

summary(results)

**5.Serial mediation (R)**

library(bruceR)

rm(list = ls())

data = read.csv(file = "C:\\Users\\Administrator\\Desktop\\mediation.csv")

data$OA=as.factor(data$OA)

data$age=as.factor(data$age)

data$RIAGENDR=as.factor(data$RIAGENDR)

data$RIDRETH1=as.factor(data$RIDRETH1)

data$DMDMARTL=as.factor(data$DMDMARTL)

data$DMDEDUC2=as.factor(data$DMDEDUC2)

data$phy=as.factor(data$phy)

data$alq101=as.factor(data$alq101)

PROCESS(data, y="OA", x="Cd", meds=c("TELOMEAN","page"),

covs=c("age", "RIAGENDR", "RIDRETH1", "DMDEDUC2", "INDFMPIR", "BMXBMI", "DMDMARTL","LBXCOT","phy","alq101"),

med.type="serial", ci="boot", nsim=1000, seed=1)

1. **Directed Acyclic Graph (R)**

library(dagitty)

library(ggdag)

dag <- dagitty:: dagitty("dag{

metals->OA;BA->OA;telomere->OA;PA->OA;

metals->PA;metals->telomere;metals->BA

metals -> telomere->BA-> OA;metals -> telomere->PA-> OA;

{age sex race marital activity alcohol BMI PIR cotinine occupation} -> OA;

{alcohol cotinine occupation} -> metals;

{age activity alcohol BMI cotinine } -> telomere;

{age activity alcohol BMI cotinine } -> BA;

{age activity alcohol BMI cotinine } -> PA;

}")

dag_tidy <- ggdag::tidy_dagitty(dag, seed = 4) %>% dag_label(labels = c(

"X" = "metals",

"Y" = "OA",

"A" = "age",

"B" = "sex",

"C" = "race",

"D" = "marital status",

"E" = "physical activity", "F" = "drinking alcohol status",

"G" = "body mass index", "H" = "the ratio of family income to poverty",

"I" = "cotinine",

"K" = "occupation ",

"O" = "OA-related medicine ",

"p"="TELO","Q"="ba","R"="PA"))

ggdag(dag_tidy, node_size = 20, text_size = 4, label_size = 1,

edge_type = "link_arc") +

geom_dag_label_repel(aes(label = label)) +

geom_dag_edges(edge_width = 0.1) +

theme_dag_blank() +

expand_plot(expand_x = expansion(c(0.1, 0.1)),

expand_y = expansion(c(0.1, 0.1)))
